# Elevated Interleukin-10 Levels in COVID-19: Potentiation of Pro-Inflammatory Responses or Impaired Anti-Inflammatory Action?

**DOI:** 10.3389/fimmu.2021.677008

**Published:** 2021-06-21

**Authors:** Hashim Islam, Thomas C. Chamberlain, Alice L. Mui, Jonathan P. Little

**Affiliations:** ^1^ School of Health and Exercise Sciences, University of British Columbia Okanagan, Kelowna, BC, Canada; ^2^ Department of Surgery, University of British Columbia, Vancouver, BC, Canada; ^3^ Department of Biochemistry and Molecular Biology, University of British Columbia, Vancouver, BC, Canada

**Keywords:** cytokine storm, SARS – CoV – 2, diabetes, immunometabolism, inflammation

## Introduction

Interleukin (IL)-10 is a pleiotropic cytokine known for its potent anti-inflammatory and immunosuppressive effects. Originally identified as a product of T helper 2 cells, IL-10 is now known to be produced by various myeloid- and lymphoid-derived immune cells participating in both innate and adaptive immunity (1, 2). A primary function of IL-10 during infection is to inhibit the host immune response to pathogens and microbiota, thereby mitigating tissue damage and immunopathology. To accomplish this, IL-10 inhibits pro-inflammatory cytokine synthesis and antigen presentation in activated monocytes/macrophages and dendritic cells, while also limiting excessive T cell activation and proliferation (1, 2). The anti-inflammatory effects of IL-10 are primarily mediated by its interaction with the IL-10 receptor (most highly expressed on monocytes/macrophages), which activates the JAK1-TYK2-STAT3 pathway leading to STAT3-mediated transcription of genes that limit the inflammatory response (1, 2). IL-10’s ability to inhibit pro-inflammatory cytokine expression also requires the inositol phosphatase SHIP1 (3) and the anti-inflammatory effects of IL-10 may specifically be mediated by its ability to induce SHIP1-STAT3 complex formation (4), thereby differentiating IL-10 signaling from other cytokines that activate STAT3 (e.g. IL-6).

## Possible Explanations for Elevated IL-10 Levels in COVID-19

A common feature and presumable cause of death among patients with severe cases of the coronavirus disease 2019 (COVID-19; caused by the SARS-CoV-2 virus) is the overproduction of pro-inflammatory cytokines arising from excessive immune cell activation (i.e., cytokine release syndrome, often referred to as “cytokine storm”) (5). The dramatic early rise in IL-10 – canonically classified as an anti-inflammatory cytokine – appears to be a distinguishing feature of hyperinflammation during severe SARS-CoV-2 infection (6) and several studies indicate that IL-10 levels predict poor outcomes in patients with COVID-19 (7, 8). Based on its well-established role as an anti-inflammatory and immunosuppressive cytokine (1, 2), the dramatic elevation in IL-10 could be interpreted as an attempt to temper hyperinflammation and prevent tissue damage. However, the concurrent elevations in IL-10 and various pro-inflammatory cytokines, and the observed relationship between elevated IL-10 levels and disease severity, suggest that IL-10 is either failing to appropriately suppress inflammation (as observed in other inflammatory conditions (9–11) or acting in a manner that deviates from its traditional role as an anti-inflammatory molecule. Indeed, one explanation for the seemingly paradoxical observation of concurrently elevated IL-10 and pro-inflammatory cytokine levels is the ability of IL-10 to act as a pro-inflammatory and immunostimulatory molecule under certain contexts (6). Another compelling and previously unexplored explanation is the potential escape of activated immune cells from IL-10’s anti-inflammatory action (i.e., IL-10 “resistance”) leading to overexuberant pro-inflammatory cytokine responses. In support of this hypothesis, we have reported resistance to IL-10’s anti-inflammatory action under hyperglycemic conditions *in vitro* (12, 13) and in individuals with type 2 diabetes (T2D) (12). Importantly, because T2D is a risk factor for increased COVID-19 disease severity and mortality (which is markedly lower with well-controlled blood glucose levels) (14), IL-10 resistance may provide a mechanistic link between hyperglycemia/T2D and adverse COVID-19 outcomes.

**Figure 1:**
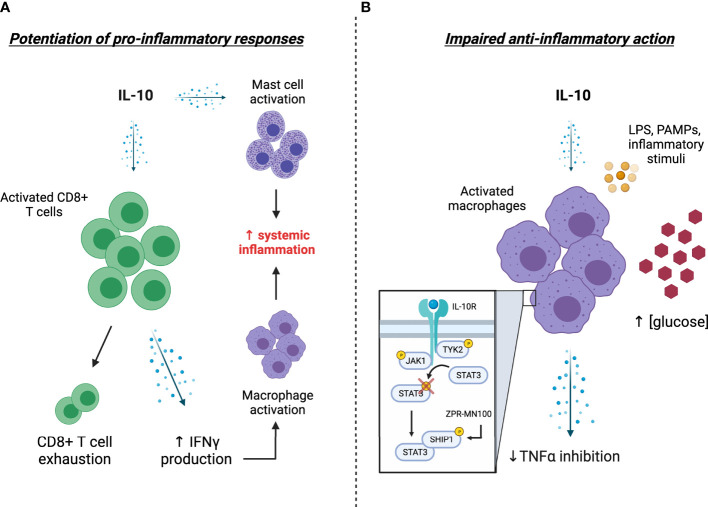
Potential explanations and consequences of elevated IL-10 levels in COVID-19. **(A)** Excessive stimulation of CD8+ T cells by IL-10 levels leads to T-cell overactivation and enhanced IFNγ levels, the latter of which further stimulates the production of pro-inflammatory factors by activated macrophages. The propagation of systemic inflammation is presumably bolstered by the IL-10 mediated activation of tissue-resident mast cells. **(B)** A hyporesponsiveness to IL-10 action (i.e., IL-10 “resistance”) impairs the ability of activated monocytes/macrophages to respond to circulating IL-10, thereby enhancing the release of pro-inflammatory cytokines such as TNFα into circulation. Mechanistically, this impairment in IL-10 action is associated with impaired STAT3 phosphorylation and appears to be driven by elevated blood glucose levels, providing a potential explanation for severe COVID-19 related outcomes in patients with diabetes. Treatment with the SHIP1 agonist ZPR-MN100 (previously known as AQX-MN100) overcomes high glucose-induced IL-10 resistance in macrophages and resolves colitis in IL-10 receptor knock-out mice, highlighting the potential of SHIP1 targeted therapeutics for combatting severe COVID-19. [Created with Biorender.com].

In this article, we present evidence supporting the non-classical pro-inflammatory effects of IL-10 as a driver of cytokine storms during COVID-19 and consider resistance to IL-10’s classical anti-inflammatory action as an alternative novel mechanism underlying elevated IL-10 levels in patients with severe COVID-19 (summarized in **Figure 1**). We also highlight the potential utility of therapeutic avenues targeting components of the IL-10 signaling pathway as a viable strategy for restoring IL-10 action in COVID-19. Given that cytokine storms arising from hyperinflammation propagate tissue damage that can eventually cause multi-organ failure and death in severe COVID-19 cases (5), a greater understanding of IL-10’s role in COVID-19 pathogenesis is warranted for the development of effective strategies aimed at combatting the current pandemic.

## Evidence Supporting the Role of IL-10 as a Pro-Inflammatory Cytokine

Although typically classified as anti-inflammatory and immunosuppressive cytokine, the effects of IL-10 are highly context-dependent and there are several scenarios where IL-10 enhances immune cell activation and proliferation causing the release of pro-inflammatory cytokines. For instance, Lauw et al. (15) were the first to demonstrate the pro-inflammatory effects of IL-10 *in vivo* during human endotoxemia. In this study, intravenous administration of recombinant IL-10 (25 µg/kg) potentiated the liposaccharide (LPS)-induced increase in IFNγ and IFNγ-dependent chemokine production in healthy humans (15). These experiments were shortly followed by studies in patients with Crohn’s disease, where subcutaneous administration of high dose recombinant IL-10 (20 µL/kg) caused an increase in IFNγ production in phytohemagglutinin-stimulated whole-blood cultures (16).

Further support for the immunostimulatory role of IL-10 comes from studies in rodent tumour models where IL-10 administration promotes proliferation and expansion of tumor-resident cytotoxic CD8+ T cells as well as IFNγ production, thereby enhancing antitumor activity (17). In line with these rodent experiments, administration of pegylated recombinant IL-10 (20 µg/kg) to human cancer patients induces systemic immune activation as reflected by elevations in various pro-inflammatory cytokines and expansion of both systemic and tumour-resident CD8+ T cells (18, 19). Collectively, these findings indicate that high doses of IL-10 can induce pro-inflammatory responses in healthy participants as well as patients with autoimmune disease and cancer.

In light of the aforementioned findings, several lines of evidence support the potential pro-inflammatory actions of IL-10 in severe COVID-19 cases. First, many of the same cytokines that are elevated with high-dose IL-10 administration in the studies discussed above (e.g. IL-4, IL-7, IL-18, IFNγ, TNFα) are also elevated in severe COVID-19 cases in conjunction with elevated IL-10 levels (7, 8, 20, 21). Of note, the rise in IL-10 levels occurs during the early stages of SARS-CoV-2 infection, thus preceding elevations in pro-inflammatory cytokines that typify cytokine storms (8). Second, plasma levels of bacterial DNA and LPS – two known pathogen-associated molecular patterns (PAMPs) that activate inflammatory signaling in immune cells – are elevated in severe COVID-19 cases (22). Although IL-10 is a potent inhibitor of LPS-induced gene expression in macrophages (23), the ability of high concentrations of IL-10 to amplify pro-inflammatory responses to LPS (15) raises the possibility that the combination of elevated IL-10 and bacterial products drives inflammation in COVID-19. Moreover, because LPS is a known inducer of IL-10 production in macrophages (1), high levels of LPS may play a causal role in the observed elevations in IL-10 during COVID-19.

Given the ability of IL-10 to potently induce T cell activation in various cancer models (17), a final piece of evidence supporting the potential pro-inflammatory actions of IL-10 in COVID-19, is the observation of overactivated CD8+ T cells despite a reduction in overall CD8+ T cell count (24). IL-10-mediated hyperactivation of CD8+ T cells despite an overall reduced cell count may also explain why some studies report functional exhaustion of T cells in severe COVID-19 cases (25) and significant inverse associations between serum IL-10 levels and T cell count (26). The propagation of systemic of systemic inflammation by CD8+ T cell derived cytokines (e.g., IFNγ) would presumably be bolstered by IL-10 mediated activation of tissue-resident mast cells (27), which are abundant in lung epithelial membranes and have been implicated in COVID-19-related inflammation (28–30). Taken together, the aforementioned findings raise the intriguing possibility that the “non-classical” pro-inflammatory actions of IL-10 may contribute to the propagation of cytokine storms in COVID-19 (6), thus warranting further research into this avenue.

## IL-10 Resistance as a Link Between Hyperglycemia/T2D and Severe COVID-19 – Related Outcomes

Another compelling and novel explanation for elevated IL-10 levels in the face of systemic hyperinflammation in severe COVID-10 cases is the potential inability of IL-10 to inhibit pro-inflammatory cytokine production and release from activated monocytes/macrophages (e.g. IL-10 “resistance”). This scenario may help explain why the existence of hyperglycemia and diabetes is linked to disease severity and mortality in patients with COVID-19 (31), and why improved glycemic control is associated with better outcomes (14). Studies from our lab were the first to demonstrate the concept of IL-10 “resistance” under hyperglycemia *in vitro* [recently replicated by an independent group (13)] and from immune cells isolated from patients with T2D (12). In our experiments, the ability of IL-10 (10 ng/mL) to inhibit TNFα production in response to LPS stimulation was reduced in whole-blood cultures from individuals with T2D as compared to healthy age and BMI-matched controls (12). A similar resistance to IL-10’s anti-inflammatory action was observed in macrophages cultured in high-glucose media suggesting that hyperglycemia was responsible for the reduced anti-inflammatory function of IL-10 in T2D (12). Mechanistically, the hyporesponsiveness to IL-10 action correlated with impaired STAT3 phosphorylation under hyperglycemia and responsiveness was restored with a small molecule activator of the inositol phosphatase SHIP1 (12), highlighting the STAT3/SHIP1 axis as a potential target for restoring IL-10 action (4). Although the concept of aberrant immune cell activation in response to high glucose is well-established, recent *in vitro* experiments indicate that exposure to high glucose also enhances SARS-CoV-2 replication in monocytes (32). This hyperglycemia-induced potentiation of SARS-CoV-2 replication in monocytes requires glycolytic flux (32), which is noteworthy (and perhaps further supportive of IL-10 resistance) because the anti-inflammatory effects of IL-10 in macrophages are typically mediated by oxidative metabolism (33).

As mentioned earlier, levels of bacterial DNA and LPS are elevated in patients suffering from severe cases of COVID-19 (22). Although one line of reasoning can interpret elevated LPS levels in COVID-19 as support for the pro-inflammatory effects of IL-10 (as above), these observations can alternatively also be interpreted as support for IL-10 resistance. Specifically, because the IL-10-STAT3 axis inhibits ~20% of LPS-induced genes (1), failure of IL-10 to inhibit cytokine production from IL-10 resistant monocytes/macrophages during endotoxemia may explain why various pro-inflammatory cytokines are elevated despite high IL-10 levels in severe COVID-19 cases.

Based on these observations, it is tempting to speculate that a similar resistance to IL-10’s anti-inflammatory action may underpin hyperinflammation in COVID-19, particularly in individuals with diabetes. Moreover, since systemic inflammation and natural killer cell activation – both of which are present during respiratory viral infections (34, 35) – can drive insulin resistance in skeletal muscle (34) and adipose tissue (36), the ensuing hyperinsulinemia/hyperglycemia may further propagate IL-10 resistance in individuals with T2D infected with SARS-CoV2. These speculations warrant further investigation to determine the contribution of IL-10 resistance to severe COVID-19 outcomes in individuals with diabetes.

## Potential Therapeutic Avenues Targeting IL-10 Signalling

If IL-10 resistance is involved in COVID-19 adverse outcomes, then recent insights into the molecular aspects of anti-inflammatory IL-10 signaling may provide clues for novel therapeutic options. Chamberlain and colleagues (4) recently reported that anti-inflammatory IL-10 signaling involves induction of a SHIP1-STAT3 complex, which translocates to the nucleus resulting in inhibition of macrophage activation and resolution of inflammatory colitis in mice. In this study, a small molecule SHIP1 agonist acted like an anti-inflammatory “IL-10 mimetic” to inhibit macrophage activation and resolve colitis in IL-10 receptor knock-out mice. In line with these observations, we previously demonstrated that small molecule SHIP1 agonists could overcome high glucose-induced IL-10 resistance in macrophages (2). Thus, it seems plausible that a loss of normal SHIP1-STAT3 complex formation might be a mechanism that contributes to IL-10 resistance and that SHIP1 agonists can circumvent this to reduce inflammation. In this manner, it is intriguing to speculate that drugs targeting SHIP1 signaling could play a role in mitigating negative consequences of cytokine storms as a therapeutic option in COVID-19.

## Conclusions

The drastic early rise in IL-10 in severe cases of COVID-19 is a distinguishing and seemingly paradoxical observation in light of IL-10’s classical role as an anti-inflammatory cytokine. The non-classical pro-inflammatory effects of IL-10 provide a plausible explanation for elevated IL-10 levels in the face of systemic inflammation (**Figure 1A**). Another novel and intriguing possibility that we have presented here is a potential “resistance” to IL-10’s classical anti-inflammatory actions, which may provide a mechanistic link between hyperglycemia/diabetes and severe COVID-19-related outcomes (**Figure 1B**). Further investigation into potential strategies aimed at counteracting the pro-inflammatory effects of IL-10 on CD8+ cells or restoration of IL-10’s anti-inflammatory action on macrophage cells may be beneficial for combatting hyperinflammation during SARS-CoV-2 infection. In this regard, small molecule SHIP1 agonists provide a promising avenue for exploration to restore anti-inflammatory IL-10 signaling.

## Author Contributions

HI, AM, and JL contributed to the conception of the work. HI and JL wrote the initial draft of the manuscript. All authors contributed to the article and approved the submitted version.

## Funding

HI holds a Natural Sciences and Engineering Research Council (NSERC) Postdoctoral Fellowship. JL is supported by NSERC (RGPIN-2019-05204) and the Michael Smith Foundation for Health Research (16890). AM is supported by the Canadian Institutes of Health Research (MOP-84539).

## Conflict of Interest

JL is volunteer Chief Scientific Officer for the not-for-profit Institute for Personalized Therapeutic Nutrition. JL holds founder shares in Metabolic Insights Inc., a for-profit company that developed non-invasive metabolic monitoring devices. AM is one of the principle founders of a start-up company (Aquinox Pharmaceuticals) to develop SHIP1 activators for the treatment of human disease. AM does not receive compensation from Aquinox, nor does she play a role in the day-to-day operations of the company.

The remaining authors declare that the research was conducted in the absence of any commercial or financial relationships that could be construed as a potential conflict of interest.
